# A Small Set of Succinct Signature Patterns Distinguishes Chinese and Non-Chinese HIV-1 Genomes

**DOI:** 10.1371/journal.pone.0058804

**Published:** 2013-03-19

**Authors:** Yan Wang, Reda Rawi, Christoph Wilms, Dominik Heider, Rongge Yang, Daniel Hoffmann

**Affiliations:** 1 Research Group Bioinformatics, Center for Medical Biology, University of Duisburg-Essen, Essen, Germany; 2 AIDS and HIV Research Group, State Key Laboratory of Virology, Wuhan Institute of Virology, Chinese Academy of Sciences, Wuhan, P. R. China; University of Amsterdam, Netherlands

## Abstract

The epidemiology of HIV-1 in China has unique features that may have led to unique viral strains. We therefore tested the hypothesis that it is possible to find distinctive patterns in HIV-1 genomes sampled in China. Using a rule inference algorithm we could indeed extract from sequences of the third variable loop (V3) of HIV-1 gp120 a set of 14 signature patterns that with 89% accuracy distinguished Chinese from non-Chinese sequences. These patterns were found to be specific to HIV-1 subtype, i.e. sequences complying with pattern 1 were of subtype B, pattern 2 almost exclusively covered sequences of subtype 01_AE, etc. We then analyzed the first of these signature patterns in depth, namely that L and W at two V3 positions are specifically occurring in Chinese sequences of subtype B/B' (3% false positives). This pattern was found to be in agreement with the phylogeny of HIV-1 of subtype B inside and outside of China. We could neither reject nor convincingly confirm that the pattern is stabilized by immune escape. For further interpretation of the signature pattern we used the recently developed measure of Direct Information, and in this way discovered evidence for physical interactions between V2 and V3. We conclude by a discussion of limitations of signature patterns, and the applicability of the approach to other genomic regions and other countries.

## Introduction

In the course of the spread of Human Immunodeficiency Virus 1 (HIV-1) around the world, the virus has evolved a number of subtypes and variants with characteristic geographical distribution [1]. The existence of such HIV-1 variants is relevant to the treatment of infected individuals and to the development of vaccines [2], [3]. Moreover, tracking of variants can be used to discover routes of infection [4]. Last but not least it is interesting to follow viral evolution and to explore the underlying evolutionary phenomena.

One of these phenomena is the founder effect, i.e. the genome of the spreading virus can retain features of the founder virus over several transmissions [5]. Another evolutionary factor is the interaction of the spreading virus with the immune systems of the infected population, which may select mutations that facilitate immune escape [6]. Further, the evolution of the virus can be affected by the mode of transmission [7], e.g. sexually, mother to child, by intravenous drug use with shared needles, by contaminated blood products or medical equipment, etc. Moreover, the network of contacts that potentially lead to transmission is important for the spread of the virus [8]. This network will in general depend on cultural, social, and technical factors. Interestingly, the speed at which the virus spreads in a population seems to be related to its evolution: the faster the spread, the lower the diversity [9].

In the case of China, there is evidence for an initial fast spread of a limited number of founder strains by intravenous drug use, contaminated blood products or medical equipment, followed by the slower mode of sexual transmission [10]. Specifically, HIV-1 subtype B' has caused an explosive epidemic in Asia via the networks of injecting drug users and unhygienic blood plasma collection (banned in China in 1996). Subtype B' can be traced back to a single founder strain existing around 1985, compared to 1966 for the most recent common ancestor of pandemic subtype B [11]. This founder strain first caused an outbreak amongst injecting drug users in Thailand and neighboring countries in the late 1980s, and then further explosive outbreaks amongst blood donors in China [12]. Therefore, according to the argument in Reference [9] mentioned earlier, we could expect that some features of the founder strains still can be detected in the HIV-1 sampled from different regions of China. In fact, Deng et al. [10] list seventeen mutations in HIV-1 genomic regions p17 and V3 that are more frequent in the South-East-Asian or Chinese strain B' than in the pandemic subtype B, from which B' has branched off.

In this work we address the following three questions: (1) Can we identify in viral sequences succinct signature patterns that are characteristic for HIV-1 from China? (2) Are these signature patterns related to HIV-1 subtypes? (3) Is it possible to understand the viability of such a pattern?

Detection of viral sequence patterns that are specific for a group of hosts requires that the studied sequence is not conserved but variable, and that sufficient amounts of sequences are available for statistical analysis. In our study we therefore first focused on the V3 loop, the third variable loop of the HIV-1 envelope protein Env, a peptide of about 35 amino acids with a sequence that correlates well with HIV-1 subtypes [1], and for which thousands of sequences are available. In order to identify patterns of amino acids that are characteristic for Chinese sequences, we apply the rule inference algorithm RIPPER (Repeated Incremental Pruning to Produce Error Reduction) [13] to multiple sequence alignments of Chinese and Non-Chinese sequences. We find that application of RIPPER to V3 sequences generates a small set of simple, subtype specific signature patterns, that distinguish Chinese from Non-Chinese sequences with an overall classification accuracy of 89%. The first of these patterns comprises just two V3 positions and is highly specific for the East-Asian B' strain, which we support by phylogenetic analysis. We then extend the analysis to the full Env protein to find possible mechanistic reasons for the viability of this pattern. To this end, we make use of the recently developed “Direct Information” approach [14] that empirically predicts pairs of interacting residues based on observed co-evolution. We conclude with a short discussion on whether the use of signature patterns lends itself to other countries, whether sequences other than V3 or Env might be suitable for this type of analysis, and what are potential sources of error.

## Results and Discussion

### Signature patterns for V3 sequences sampled in China

#### Inference of signature patterns

Application of the RIPPER [13] algorithm for rule inference to an aligned set of 1047 Chinese and 1288 Non-Chinese V3 sequences (see Materials and Methods and supplementary information) resulted in the following 14 signature patterns:

1. (×22 = W) and (×14 = L)  = >Chin = TRUE (257/8)
2. (×24 = R)  = > Chin = TRUE (335/41)
3. (×20 = Q) and (×12 = I) and (×21 = T) and (×5 = G)  = > Chin = TRUE (103/17)
4. (×20 = Q) and (×12 = I) and (×21 = T) and (×26 = G) and (×10 = K)  = > Chin = TRUE (120/20)
5. (×22 = W) and (×33 = Q) and (×10 = K) and (×14 = I)  = > Chin = TRUE (57/5)
6. (×20 = Q) and (×35 = Y) and (×33 = K) and (×24 = K)  = > Chin = TRUE (20/1)
7. (×21 = S) and (×20 = R)  = > Chin = TRUE (28/1)
8. (×20 = Q) and (×12 = I) and (×26.5 = Q)  = > Chin = TRUE (7/0)
9. (×21 = T) and (×35 = Y) and (×10 = K) and (×24 = A) and (×12 = V)  = >Chin = TRUE (11/1)
10. (×23 = H) and (×26.5 = R)  = > Chin = TRUE (19/2)
11. (×21 = V) and (×5 = S)  = > Chin = TRUE (26/5)
12. (×22 = L) and (×10 = K) and (×14 = L) and (×24 = T)  = > Chin = TRUE (6/0)
13. (×22 = L) and (×24 = A) and (×18 = W)  = > Chin = TRUE (5/0)
14.  = >Chin = FALSE (1341/154)

E.g. the first signature pattern reads: “If a V3 amino acid sequence at position 22 has a W and at position 14 has an L, then it is a sequence from China.” Positions 22 and 14 refer to the positions of amino acids 22 and 14 of the HXB2 reference sequence in the multiple sequence alignment of all V3 input sequences. This pattern applies to 257 sequences, amongst which we have 8 false positives, i.e. sequences not from China. The other patterns can be interpreted analogously. The fourteenth rule “⇒Chin = FALSE” says that if none of the previous thirteen patterns has been found in a sequence, it is a Non-Chinese sequence. This last rule applies to 1341 sequences in the data set, including 154 false positives, i.e. sequences from China that do not carry any of the previous patterns. The signature patterns are succinct, including one to five sequence positions that are usually discontinuous (exception: pattern 7). All positions in the patterns have corresponding positions in the reference sequence HXB2, except in patterns 8 and 10 where the fifth gap position after HXB2 position 26 (indicated by ×26.5) in the V3 alignment is occupied by Q and R, respectively, in some Chinese sequences. The false positive rates range from 0% to 19%, while the overall false negative rate (see rule 14) lies at 11%. The fourteen signature patterns distinguish Chinese from Non-Chinese sequences with an overall classification accuracy of 89%.

#### Sequence positions in signature patterns tend to have higher entropies

**Figure 1:**
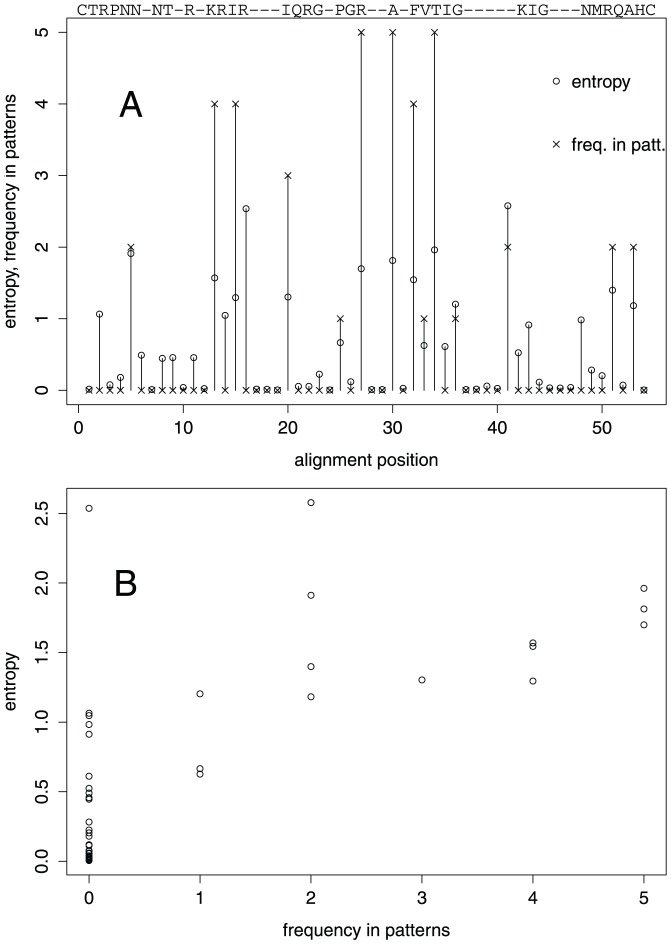
Shannon entropy and frequency of sequence positions in signature patterns. (A) For all positions of the V3 sequence alignment the entropy is shown (circles) together with the frequency with which the positions occur in the thirteen first signature patterns for Chinese sequences (crosses). The aligned HXB2 reference sequence is printed above the plot for orientation. (B) The entropy is plotted versus the frequency of occurrence of sequence positions in V3 signature patterns for Chinese sequences.

Of 54 alignment positions, only 14 positions occur in the patterns, with some occurring several times. This in turn means that 40 positions are non-informative, maybe because they are conserved, or non-conserved and non-distinctive, or redundant. To distinguish between these possibilities, we computed the sequence entropy for all alignment positions and plotted it against the frequency of occurrence of alignment positions in the first thirteen signature patterns (Figure 1A). Figure 1B shows a positive correlation between the frequency of occurrence of alignment positions in signature patterns, and the sequence entropy at these positions (Spearman's *p* = 0.71). The rank correlation is significantly different from zero (*p* = 1.8˙10^−9^). The Figure shows that the sequence positions that do not turn up in the signature patterns are mostly conserved in the alignment, while there is a tendency for the positions with higher entropy to occur more often in the patterns. There is one drastic outlier with respect to the latter observation, namely alignment position 16 (in HXB2 R at position 13) with a top ranking entropy of about 2.5 bit but not occurring in the Chinese sequence patterns at all. This position has a similar distribution of amino acids in the Chinese and the Non-Chinese group.

#### Signature patterns are associated with HIV-1 subtypes

**Figure 2:**
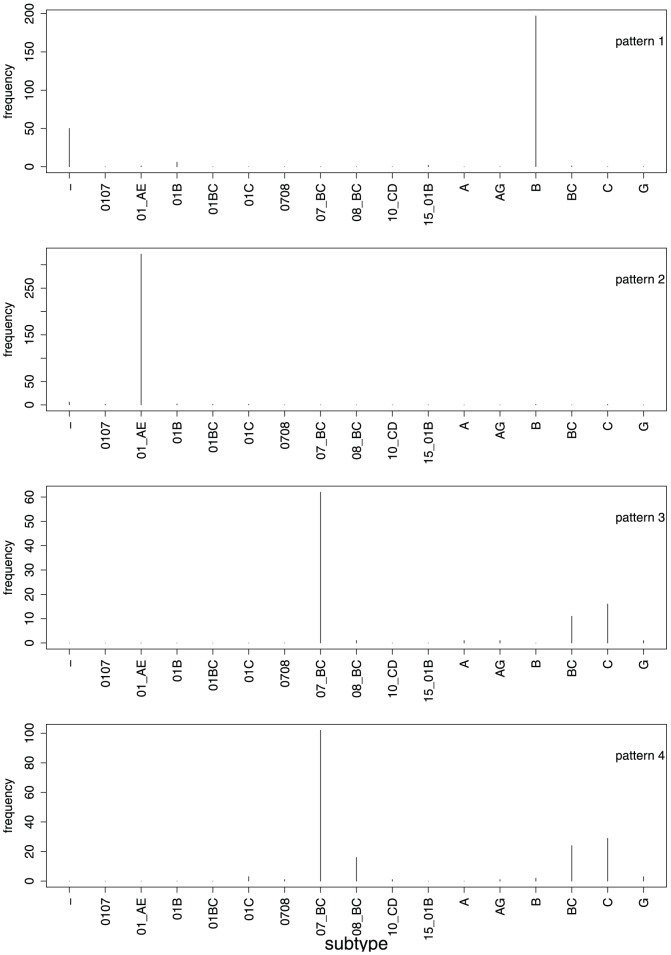
Signature patterns and subtypes. The distribution over subtypes is shown for the number of V3 sequences that conform with each of the signature patterns with more than 100 positives (patterns 1 through 4). Only subtypes are listed for which the number of sequences from China in the data set is . Sequences without subtype annotation are assigned to the leftmost class (“-”).

An HIV-1 subtype can be considered a set of HIV-1 strains that share an extended and exclusive signature pattern [1]. The likely explanation for sharing such patterns is common ancestry. The same reasoning could apply to the succinct signature patterns for Chinese sequences derived above: They could be modifications inherited from common ancestors. If this reasoning is correct, we should expect that the signature patterns are subtype specific. In other words, a signature pattern could be characteristic for a phylogenetic branch within a subtype. Figure 2 shows for the four strongest signature patterns (all patterns with more than 100 positives) that indeed the Chinese signature patterns have a strong association with subtype.

Pattern 1 is almost exclusively associated with subtype B (including the East-Asian variant B'), with only a few positives in recombinant forms involving subtype B.

Pattern 2 is clearly related to subtype 01_AE: of 330 sequences of known subtype that have an R at position 24, 323 are of subtype 01_AE. If we consider that position 24 has a relatively high entropy and occurs often in the patterns (Figure 1A), this close association of the pattern with subtype 01_AE could be due to some functionally important interaction of R at position 24 with the characteristic sequence background of 01_AE.

Patterns 3 and 4 are present in subtype C and some (though not all) recombinant forms involving subtype C. Since the two patterns are non exclusive and their distribution over the subtypes are seemingly similar, one might ask whether they are different at all. In fact, we find that 71 sequences show both signature patterns simultaneously, while 22 sequences have only pattern 3 and 111 sequences have only pattern 4. Thus, the sets complying with the two patterns are not exactly the same and joining the two patterns would result in a loss of many positives. Another strategy could be to drop the last part of both patterns and use only the first three positions (×20 = Q and ×12 = I and ×21 = T). This would lead to a total of 192 Chinese sequences complying with this pruned pattern, but the number of false positives would increase to 75. Hence, the use of the two separate rules can be justified by the higher accuracy.

Note that for several subtypes the intersection with the sets of sequences conforming with one of the four first signature patterns is empty. For instance, there are 127 sequences of subtype D (from Eastern and Central Africa) in the V3 input set, but none of these conforms with any of the first four patterns. This indicates that despite the brevity of the patterns, they are far from being uniformly randomly sampled but specific to subtypes.

### Analysis of signature pattern 1

Given the high variability of V3, it seems unlikely that these short signature patterns are fixed in the Chinese viral population independently of other parts of the virus. It is more probable that these signature patterns are tips of icebergs of larger patterns of positions that are functionally coupled. Another possibility is that the patterns are due to specific features of the host population, e.g. specific HLA types could select strains with certain immune escape mutations [6], or there could be a frequent CCR5 polymorphism in East Asia that selects pattern 1 [15]. In the following we will try to characterize signature pattern 1 (×14 = L and ×22 = W) more comprehensively, narrow down the number of possible explanations of this pattern, and relate it to molecular features that may be relevant to function.

#### Signature pattern 1 is characteristic for Thai/Chinese subtype B'

We focus on pattern 1 because it covers many sequences and is remarkably accurate: Of 257 positives, only 8 are not from China. A Fisher's exact test shows that the association between sampling in China and pattern 1 is highly significant (*p*<2.5˙10^−16^). Notably, amongst the 8 false positives there are 5 from Thailand, including the earliest sample from the year 1992. As mentioned earlier and shown by the top panel of Figure 2, almost all sequences of known subtype complying with pattern 1 are of subtype B. When we connect these two facts, it seems likely that pattern 1 marks the a variant of pandemic subtype B named Thai-B or B' [16] that is relatively frequent in China [10]. In fact, L14 and W22 were amongst the four positions in V3 found to be specific for B' in Reference [10].

#### Pattern 1 compatible with stabilizing contacts within V3 and with co-receptor pocket

**Figure 3:**
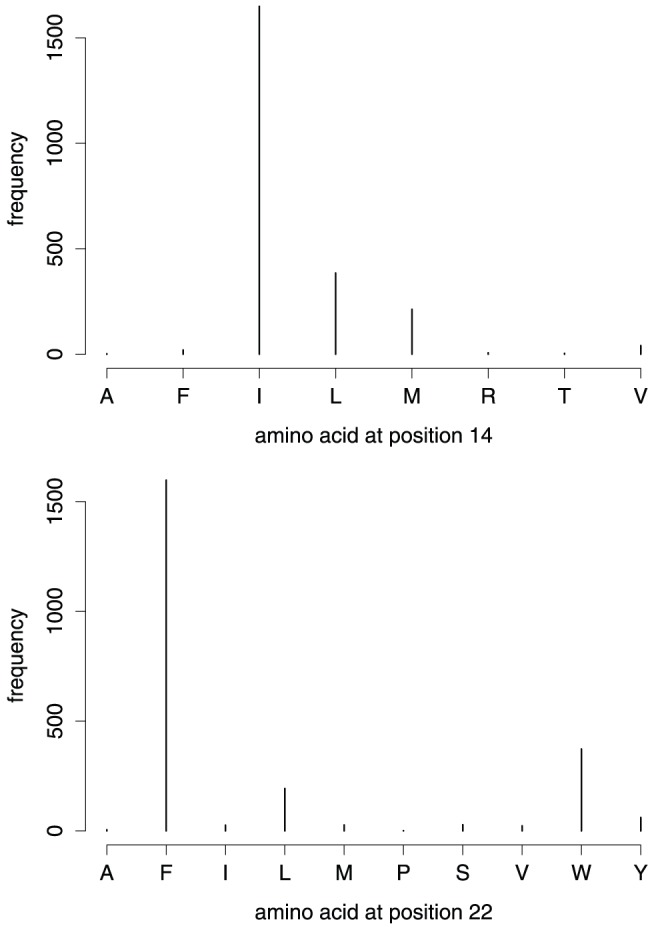
Amino acids at positions 14 and 22. The positions covered by signature pattern 1 have a preference for I and F, or, more generally, for hydrophobic amino acids at position 14 and aromatic residues at position 22. Frequencies from all Chinese and Non-Chinese samples in sequence data set.

**Figure 4:**
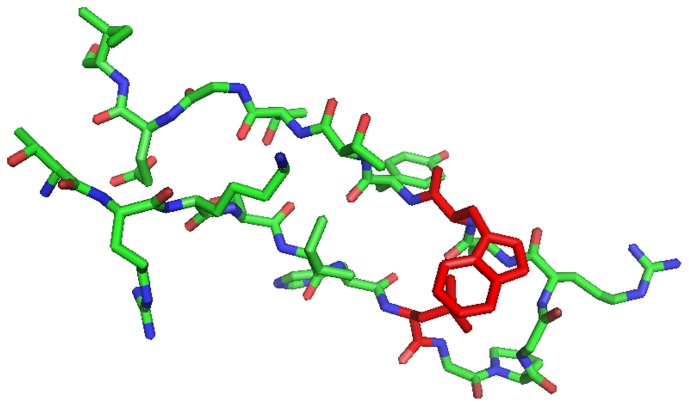
Structure of the V3 loop around pattern 1. In the NMR structure of the V3 loop by Rosen et al. [17] (PDB entry 2esx) the residues at HXB2 positions 14 and 22 have been mutated with pymol (DeLano Scientific LLC, San Francisco, CA, USA) into L and W, respectively (red), leaving the rest of the loop unchanged. The two modeled side chains are touching each other.

Positions 14 and 22 generally have a strong preference for hydrophobic and aromatic amino acids, especially I and F, respectively (Figure 3). Experimental structures [17], [18] of the V3 loop suggest that a direct hydrophobic contact of the two residues could be possible (Figure 4). In these structures, the two residues are spatial neighbors from opposite strands of the V3 -hairpin. The side chains are pointing to the same side of the hairpin so that it is also imaginable that the two residues jointly bind to another site [19], either in gp120 or at the co-receptor CCR5. It is remarkable that position 14 prefers hydrophobic but not aromatic residues while position 22 prefers aromatic residues (Figure 3). This supports a specific interaction with a binding partner, such as CCR5. In fact, the region between the second extracellular loop of CCR5 (ECL2) and parts of transmembrane helices of CCR5 contain pockets lined by hydrophobic and aromatic residues [20]–[22]. Moreover, these pockets are targeted by the CCR5 blocking drugs such as Maraviroc [23], Aplaviroc [22], or Vicriviroc [24] which all have hydrophobic and aromatic functional groups in arrangements similar to the side chains of I/L14 and F/W22.

#### No conclusive evidence for immune escape as cause for pattern 1

We have mentioned the possibility of an HLA epitope covering the region from L14 to W22. Insertions or deletions in this region are rare in our data set (HXB2 being one of the few exceptions), so that for more than 98% of all sequences the peptide from L14 to W22 has a length of seven amino acids. This length fits into typical MHC I binding peptides [25]. Therefore L14 and W22 could be CTL escape mutations for an HLA type that is frequent in East Asia. Unfortunately, none of the Chinese V3 sequences in our data set is annotated with HLA information. There is some experimental evidence [26] for a possible immune escape of HIV-1 with pattern 1 in carriers of HLA type A*30, and also for a reduced recognition by HLA type A*02, the latter being relatively frequent in Han Chinese [27]. To predict the effect of I14L and F22W mutations, we used NetMHC-3.0 [28]. We screened several V3 sequences with and without I14L and F22W and found consistently a strongly decreased recognition of the nonamer with L at second position and W at eighth position by A*0250, but not for other HLA types offered by NetMHC. According to these predictions, the main effect comes from the I14L mutation, possibly destroying the anchor to the MHC I molecule. Since MHC II can be important for virus control, too [29]–[32], it is theoretically possible that signature pattern 1 is associated to immune escape by reduced MHC II binding. We have therefore used Epitope Location Finder (http://www.hiv.lanl.gov/content/sequence/ELF/epitope_analyzer.html. Accessed 2012 Oct 1) to check whether L14 or W22 could be anchor sites for MHC II epitopes, and we found that indeed position 14 is an anchor sites for some HLA II types (DRB1*0101, DRB1*0401, Cw*1801) so that I14L could be an escape mutation. However, according to the Allele Frequencies database (http://www.allelefrequencies.net. Accessed 2012 Aug 21) HLA II types DRB1*0101 and DRB1*0401 are less frequent in China compared to Europe or North-America, and Cw*1801 is more sparsely documented. In summary, the available data does not point clearly to HLA escape as main cause for pattern 1, but given the sparse HLA data associated with HIV-1 genomes from China, we cannot also not rule out that HLA escape plays a role.

#### Trp in pattern 1 selected more strongly than Leu

The above prediction for MHC I indicated that L14 and W22 somehow contribute differently to pattern 1. To investigate this further, we retrieved from the Los Alamos database Chinese clonal V3 sequences of subtype B, with the further requirement of more than one sequence per patient. For the resulting 432 sequences from 61 patients the amino acid frequencies at HXB2 positions 14 and 22 were counted. If L14 is more important for replication fitness than W22, we should see more often L14 than W22, and vice versa. It turned out that W22 was almost completely conserved in all patients (97%) with a small contribution of L at this position (2%) and a mixture of other, mostly hydrophobic, amino acids. Position 14 showed a greater variability with 74% L, 22% I, 3% M, and 1% V. This observation suggests much stronger constraints on W22 than on L14. This view is supported by independent results on the ratio of synonymous and non-synonymous mutations in the Env gene [33], showing that W22 is under strong positive selection pressure, while L14 is not. On the other hand, despite the variability at position 14, we never see an aromatic and hydrophobic amino acid there (F, W), and therefore cannot exclude negative selection.

#### Signature pattern 1 is part of a larger pattern

Since the variability of position 14 is much higher than that of position 22 it seems unlikely that the conservation of W22 can be explained exclusively by the stabilizing hydrophobic contact with L14 described above (Figure 4). Similarly, it is unlikely that another specific residue in V3 can explain fixation of W22 because this would possibly have led to a different signature pattern. Nevertheless, we tested the latter hypothesis by applying RIPPER to all alignment columns except HXB2 position 14. We obtained 16 patterns with only the first involving W22, namely “(×22 = W) and (×26.5 = Q) chin = TRUE (204/10)”. Q at HXB2 position 26.5 has also been found as B' signature mutation by other methods [10]. In comparison to the original pattern 1 the positives dropped from 257 to 204, while the false positives slightly increased from 8 to 10, i.e. the new pattern 1 is weaker than the original one. This result does not support a simple model of fixation of W22 solely by other residues within V3.

#### Support of pattern 1 by phylogenetic analysis

**Figure 5:**
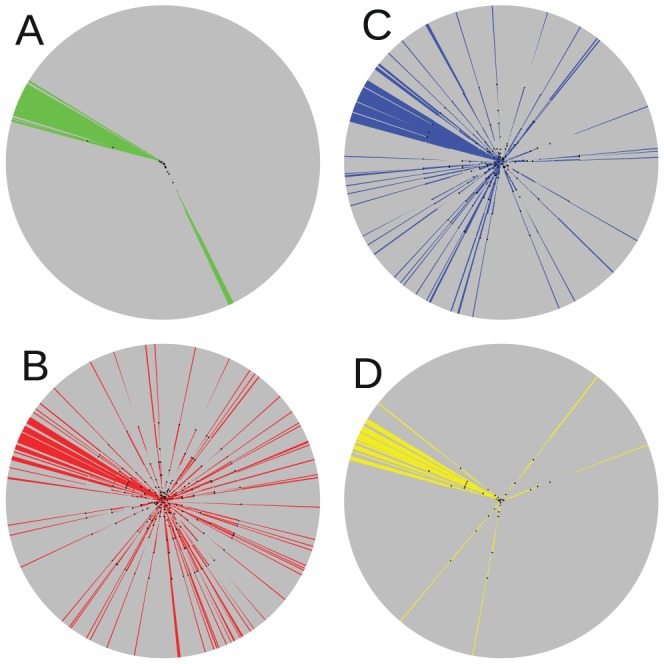
Phylogenetic fan tree of subtype B/B' Env sequences. The four panels show four times the same tree with coloring highlighting different properties. A: sequences from China in green; B: sequences with L14 in red; C: sequences with W22 in blue; D: sequences carrying signature pattern 1 (L14 and W22) in yellow.

Phylogenetic analysis is a reference way of clustering evolutionary data. Hence we have tested whether sequences carrying signature pattern 1 form a cluster that can be explained by retroviral evolution. To this end we have computed a maximum likelihood phylogenetic tree for a set of 954 subtype B/B' amino acid sequences of HIV-1 Env, shown in Figure 5 (see also error considerations in Text S3 and Figure S5). Most of the Chinese subtype B sequences clearly form a large B'-cluster around 10 hours in the fan tree in Figure 5A. Most of the sequences with L14 *or* W22 sequences are lying in this cluster, but a fair amount of these sequences are scattered over the whole tree (Figures 5B and C). If we combine both positions to signature pattern 1 (L14 *and* W22, Figure 5D), the overwhelming majority of sequences with this pattern lie around 10 hours in the Chinese cluster. Thus, signature pattern 1 is consistent with retroviral evolution of subtype B/B'.

### Signature patterns for Chinese Env of subtype B

#### Application of RIPPER to full Env does not lead to V3 patterns

Now we come back to possible mechanisms stabilizing signature pattern 1. As outlined above, stabilization solely within V3 is unlikely. This suggests that the conservation of signature pattern 1, and especially of W22, could be due to a stabilizing pattern in the Chinese subtype B' that extends beyond V3 and of which W22 is a part. For instance, W22 could interact with other residues in Env [19]. Thus, we applied RIPPER to a multiple sequence alignment of Env subtype B sequences to find patterns that distinguish East Asian (including Chinese and Thai, class label “CNTH”) and Non-East Asian sequences and that possibly include W22 in V3. We obtained the following patterns (HXB2 Env numbering):

1. (×553 = R)  = > CNTH = TRUE (76/6)
2. (×170 = -) and (×275 = S)  = > CNTH = TRUE (8/2)
3. (×11 = L) and (×10 = W)  = > CNTH = TRUE (9/1)
4. (×372 = A) and (×277 = I)  = > CNTH = TRUE (4/1)
5. (×854 = T) and (×195 = N)  = > CNTH = TRUE (4/1)
6. (×24 = T) and (×62 = K)  = > CNTH = TRUE (2/0)
7. (×396 = K) and (×146 = S)  = > CNTH = TRUE (5/2)
8. (×145 = A) and (×350 = K)  = > CNTH = TRUE (2/0)
9.  = > CNTH = FALSE (1846/4)

None of the patterns includes W22 in V3 (or W317 in HXB2 numbering of full Env). However, the new pattern 1, referring to HXB2 Env position 553 (in gp41), of this small set is remarkably simple and powerful. Hence, this seems to be a good signature pattern for subtype B', too. This conclusion is confirmed by phylogenetic analysis (see Figure S1). Compared to pattern 1, each of the other positive patterns classifies much fewer sequences.

#### Physical interactions within Env patterns cannot be ruled out

Since the above Env patterns for subtype B' do not include W317, we went one step further and used RIPPER to infer from the same data patterns with position 553 removed from the process, thus forcing the algorithm to search for small and strong patterns that do not include this position. We obtained:

1. (×167 = T) and (×792 = A)  = > CNTH = TRUE (56/0)
2. (×275 = S) and (×317 = W)  = > CNTH = TRUE (21/4)
3. (×11 = L) and (×12 = T)  = > CNTH = TRUE (9/1)
4. (×170 = -) and (×388 = S) and (×459.18 = N)  = > CNTH = TRUE (4/0)
5. (×266 = S) and (×731 = G)  = > CNTH = TRUE (6/2)
6. (×347 = M) and (×401 = W)  = > CNTH = TRUE (2/0)
7. (×141 = L) and (×192 = M)  = > CNTH = TRUE (2/0)
8.  = > CNTH = FALSE (1856/8)

The second of the resulting Env patterns indeed contains W317. Note that in this set we have two major patterns, 1 and 2. The first pattern joins positions 167 in V2 and 792 in gp41, the second positions 275 in the gp120 outer domain and 317 in V3. In the absence of a high resolution structure of a trimeric spike, it is difficult to say whether positions 167 and 792 physically interact. However, a recent cryo-electron-microscopy structure of the trimeric spike shows that V1/V2 and V3, and gp41 contribute to interprotomer contacts [34]. Hence, we cannot exclude that the two positions in pattern 1 somehow communicate. For pattern 2: judged from the structure of the CD4 bound gp120 [18], S275 and W317 have a distance of more than 6 nm, so that no direct interaction within the same monomer seems possible without major rearrangements. Again, it cannot be excluded that in the trimeric spike there is an interaction between these two positions residing on neighboring gp120 monomers. In summary, while the V3-based pattern 1 of L14 and W22 is compatible with a direct interaction between the two residues, other observed signature patterns cannot be explained as easily with the current knowledge about Env. However, the patterns are in good agreement with phylogenetic clustering (see Figures S1, S2, S3, S4).

#### Use of Direct Information to test for physical interactions in Env

Recently, the so-called “direct information” (DI) has been introduced [14], a method to extract from multiple sequence alignments pairs of sites that co-evolve and are likely to interact physically. Since co-evolution of interacting sites is one of the possible explanations for the observed signature patterns, it is interesting to check whether pairs of alignment positions involved in signature patterns also have high DI values and are thus likely to interact. We have therefore computed the DI values for all residue pairs in Env based on a multiple sequence alignment of subtype B Env, and specifically looked for occurrence of positions 309 (14 in V3) and 317 (22 in V3) in such pairs. Since the probability for physical interaction has empirically been found to decline with decreasing DI [14], we focus here on the top ranking 200 of the more than 250 000 DI values for residue pairs in Env. Only DI values between residues with sequence distance residues are reported.

#### DI suggests many physical interactions in Env involving V2 or V3

**Figure 6:**
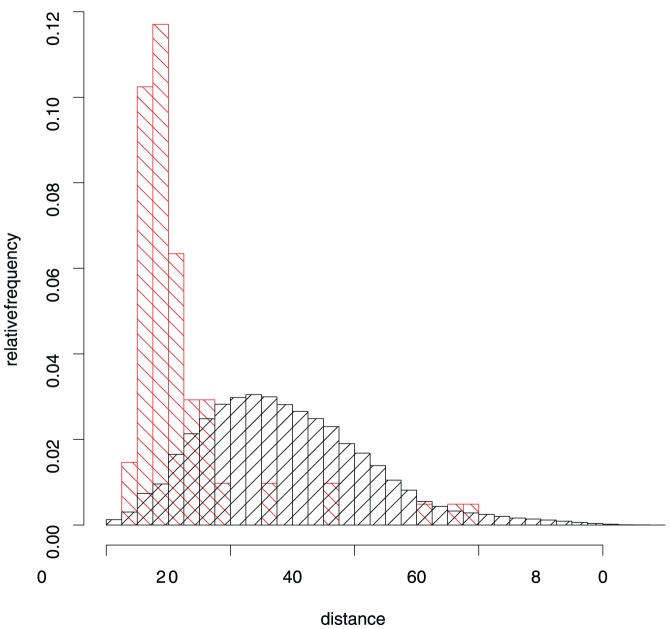
Histogram of distances in HIV-1 gp120. The black histogram shows the relative frequency of C–C distances in Å in the crystal structure of gp120 [18]. The red histogram gives the subset of these distances for residue pairs with the 200 highest values of Direct Information.

First we checked whether high ranking DI values in the case of Env coincide with short distances. Figure 6 shows that this is the case: for high DI values we have a strong enrichment of relatively short distances. However, it should be noted that for the 200 highest DI values we could only evaluate 82 distances since the structures of many molecular parts involved in low DI pairs have not been resolved experimentally. Residues from gp41 have the highest absolute number (134) of occurrences in the list of high-DI pairs, followed by the outer domain (75), V2 (45), V3 (43), and smaller numbers for the remaining parts. If we consider the relatively small sizes of V2 and V3, these two regions of Env are involved in an amazing number of putative interactions. This high number may partly be due to the fact that these two variable regions are also most visible to the DI analysis, which relies on observed mutations.

#### DI supports direct physical interaction within V3 signature pattern 1

**Figure 7:**
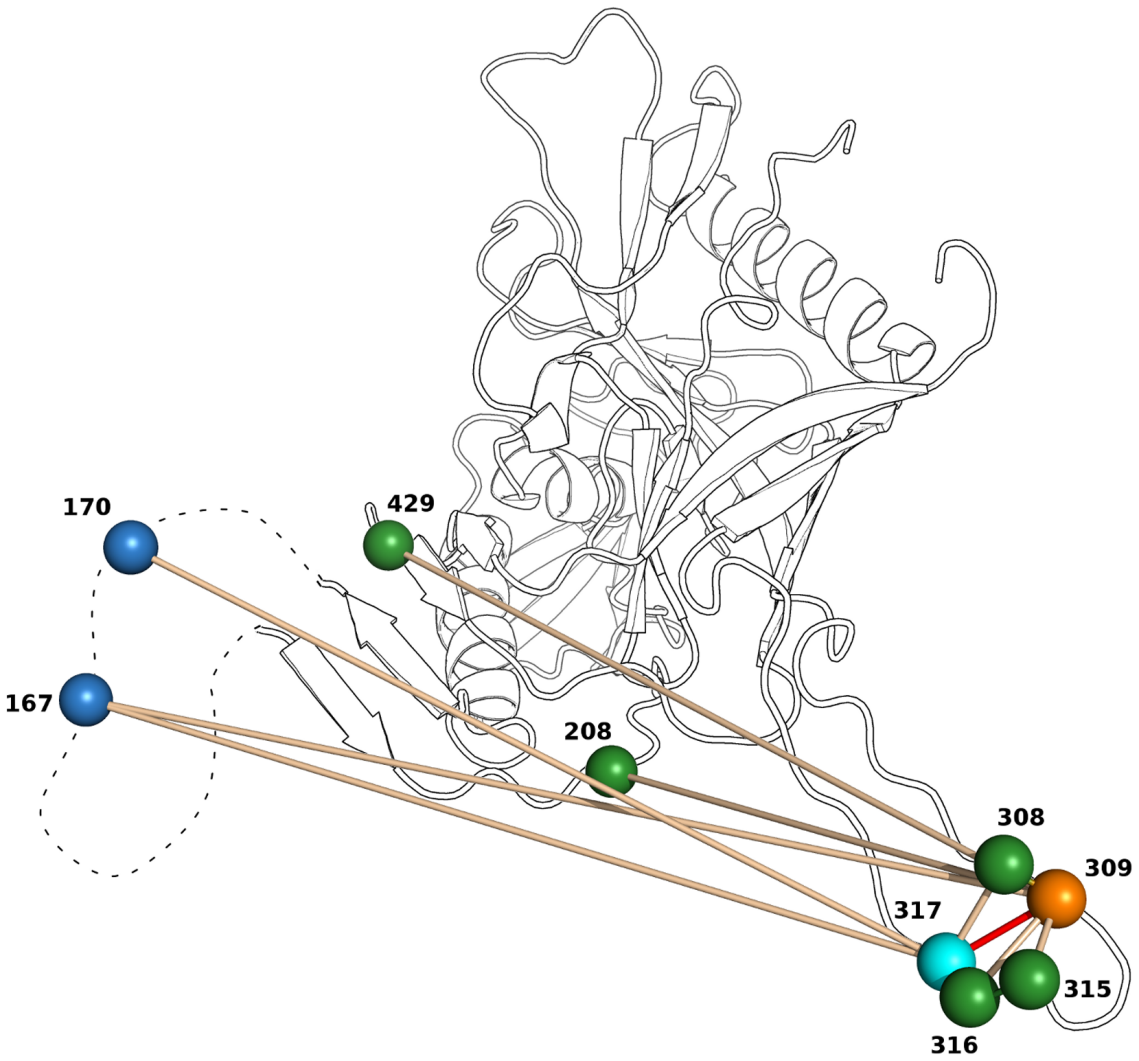
Putative interactions of signature pattern 1. The x-ray structure of gp120 [18] is shown as a cartoon with high ranking DI pairs involving signature pattern residues 309 (orange) and 317 (light blue) marked by spheres connected by bars. Most of these pairs cluster around the two residues in the V3 loop on the lower right. In addition, there are high ranking DI pairs with residues 167 and 170 (dark blue) in V2 (dashed, no x-ray structure available), and with residues 208 and 429 in the inner domain 2 and bridging sheet, respectively.

Figure 7 depicts potential interactions of signature pattern 1 residues 309 and 317 with other residues as inferred from top-200 DI values. The highest DI values involving positions 309 and 317 are to residues within V3. On one hand this makes sense as these residues are in close proximity and thus likely to interact physically. Moreover, they form a patch that possibly binds as such to other parts of Env and of the co-receptor, which imposes further constraints that may be reflected by co-evolution evaluated by DI. On the other hand, and as mentioned above, the high variability of this region makes it particularly easy for DI to detect such interactions.

#### DI and physico-chemical properties hint at V2–V3 interaction

**Figure 8:**
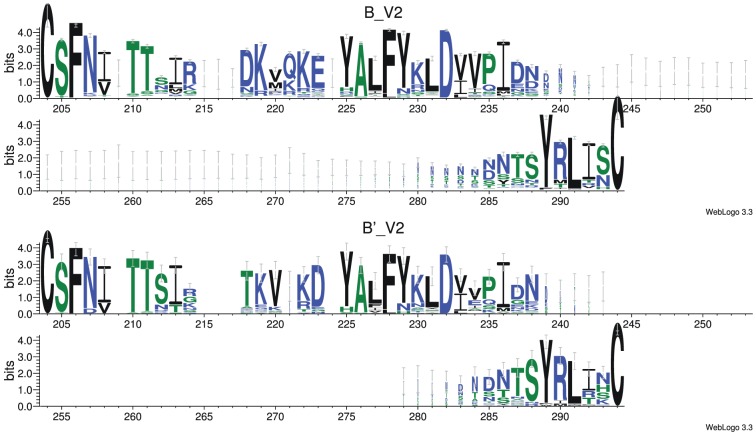
Sequence logos of V2 for subtype B (top) and B' (bottom). Numbering according to alignment positions. HXB2 positions 167 and 170 correspond to alignment positions 218 and 221, respectively. Letter height gives information content with error bars indicating estimated 95% confidence interval.

Figure 7 also shows three putative interactions between signature pattern 1 residues in V3 and residues 167 and 170 in V2 [35] that are amongst the top 200 DI values in Env. Note that residues 167 and 170 also both occur in the second set of Env signature patterns. An interaction between V2 and V3 would be in agreement with recent experiments [36], [37]. In the following we try to interpret these high-DI pairs in terms of physico-chemical properties. When we inspect V2 for Chinese B' and Non-Chinese subtype B (Figure 8), we find as most conspicuous differences between B and B' the residues at the two V2 positions 167 and 170. At position 167 (218 in Figure 8), Non-Chinese sequences have mainly a negatively charged D while Chinese sequences have a neutral T, and at position 170 (221 in Figure 8) Non-Chinese sequences have mainly a basic amino acids (K, R) or a polar Q, while Chinese sequences have a gap. Thus, it is possible to understand the double mutation D167T, K/R/Q170- as a way of maintaining the net polarity in this region, which is typically positive (2–3 positive charges, 1–2 negative charges).

#### Trp in V3 signature pattern 1 could form stabilizing cation- interaction with V2

We had marked earlier that position 317 (V3 position 22) is usually occupied by an aromatic residue, mostly F and in B' W. Why should an aromatic amino acid interact with the positively charged region in V2 as suggested by DI (Figure 7)? This interaction could be explained by cation- interactions between the cationic V2 region and the -electron system of the aromatic F or W [38], [39]. There is another concerted change that is consistent with this interaction. Remember that the Chinese B' has a gap at position 170 (Figure 8) and typically the large aromatic residue W at 317, while in the Non-Chinese we have a larger residue (K, R, Q) at 170 and a smaller aromatic F at 317. This pattern of compensatory mutations K/R/Q170- and F317W is consistent with a conserved volume in a spatial region formed by a V2–V3 contact. Note that such interactions between V2 and V3 are in agreement with recent experimental data [34], [36], [37] and a model that such interactions are necessary for control of functional conformations of gp120 [40].

### Scope and problems of signature pattern analysis

As pointed out earlier, the epidemiology of HIV-1 in China has special features that probably make it different from that in some other countries and allow for detection of distinct signature pattern for Chinese sequences, as demonstrated above. For smaller countries with higher ratio of transmissions between different countries to transmissions within that country such signature patterns may not be as accurate. To test this we tried to infer signature patterns in the same way, but this time with V3 sequences sampled in Germany vs. V3 sequences sampled in other countries. We obtained the following six patterns:

1. (×20 = R) and (×24 = A) and (×16 = I)  = > DE = TRUE (433/183)
2. (×12 = I) and (×2 = T) and (×21 = S)  = > DE = TRUE (21/5)
3. (×16 = M) and (×27 = D) and (×24 = T)  = > DE = TRUE (27/4)
4. (×16 = M) and (×5 = G)  = >DE = TRUE (17/4)
5. (×12 = I) and (×36 = -)  = > DE = TRUE (11/0)
6.  = > DE = FALSE (1498/406)

The by far dominant positive pattern 1 here has 42% false positives, while the negative pattern 6 has an error of 406/1498 = 27%. This result is in agreement with our hypothesis of a less well defined viral population in Germany. For other countries that may be more secluded, have large outbreaks, etc. this may look different.

Another interesting question is whether other regions of Env or of the whole genome of HIV-1 are also well-suited for the extraction of signature patterns. The above results for patterns that extend beyond V3 (e.g. R553 in gp41) already suggests that other parts of Env may also be informative, and that signature patterns can be spread over a long stretch of sequence. A first survey of other parts of the genome (V2, Gag, PR) showed qualitatively similar results. However, for the specific problem of Chinese signature patterns, Env and especially V3 may currently be indeed one of the best choices for the trivial reason that a relatively large number of Env/V3 sequences from China is available.

Signature pattern analysis by rule inference is essentially a statistical technique that heavily relies on the quality of the input. This means in particular that sequence input has to be representative of the population under investigation, and that the multiple sequence alignment should be reliable. Both factors, sequence sample and alignment can be critical for the detection of signature patterns. For instance, we found that while the muscle alignment software worked well on V3 sequences, it destroyed a pattern in V2 that was clearly present in the original alignment retrieved from the Los Alamos HIV database (for an extended analysis of statistical errors of this and other types see Text S3). In general, we found that the patterns and their number can change depending on sequence sample and alignment method, but that strong and short patterns as V3 signature pattern 1 will often be robust. While robust detection of patterns is a matter of sampling and aligning representative sequences, we can also expect real changes of patterns over time due to the continuing evolution of HIV-1.

Another technical question is whether we might be able to increase classification accuracy with other, more advanced machine learning techniques. Therefore we trained a random forest on the same data, achieving an accuracy of 91% in recognizing Chinese V3 sequences. However, the small improvement over rule inference (89% accuracy) has to be paid for by the loss of transparent output in the form of signature patterns. In summary, we found rule inference from multiple sequence alignments a fast and easy to use method that can provide instructive results for epidemiology and analysis of molecular evolution.

## Materials and Methods

All sequences and their associated information (for instance HIV-1 subtypes) were retrieved from the Los Alamos HIV sequence database (http://www.hiv.lanl.gov/. Accessed 2012 Oct 1). The V3 data set for the inference of rules for Chinese sequences has been compiled by combining two sets, a set of V3 sequences published as supplementary information to Ref. [41] with the small number of sequences sampled in China removed, and another set of sequences sampled in China. Duplicated sequences and sequences with non-canonical amino acid codes were removed from the two sets. The HXB2 sequence was included as a reference for the numbering of amino acids (number 1 corresponds to first Cys residue). In this way we obtained a rather balanced combined data set of 1047 Chinese and 1288 Non-Chinese sequences. By “Chinese” sequences we mean sequences annotated with nationality label CN in the Los Alamos database, and accordingly, by “non-Chinese” we mean sequences annotated there with a different nationality label. Note that it cannot be excluded that a sequence labeled CN comes from a non-Chinese or vice versa. However, many of the sequences have been generated in studies and reported in articles that describe patients; based on these published data, the mislabeling of sequences seems to be not a relevant problem.

The combined set was re-aligned with muscle [42], version 3.5, with default parameters (alignment provided as Text S1). Additional information such as subtype, sampling country, etc. were extracted from the sequence headers. R-package bio3d [43] was used for sequence processing and analysis. The command “entropy” in bio3d was used to compute Shannon entropies *S_j_* for alignment positions *j* based on a 22-letter alphabet, including the canonical 20 amino acid letters, the gap symbol “-”, and “X” (the latter being not used here):



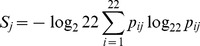
(1)


with the relative frequency *p_ij_* of letter *i* at alignment position *j*.

Signature patterns characteristic for Chinese sequences were inferred from the alignment with the program JRip [44] in RWeka [45], a package of R [46] available from http://cran.r-project.org (version 0.4–12, 2012-08-20). JRip implements RIPPER [13], an algorithm suitable for the fast inference of rules from large data sets. Further statistical analyses were performed with R [46], version 2.14.1. An example R-script that generates the described signature patterns in V3 is provided as Text S2.

For the assessment of the stability of signature pattern 1, we extracted from the Los Alamos HIV sequence database all clonal sequences of subtype B containing V3 and having been sampled in China with more than one clonal sequence per patient (432 sequences from 61 patients). Sequences were cut down to the V3 motif and, together with the V3 sequence of HXB2, realigned with muscle.

Direct information [14] values were computed with a python script that gave the same results as the reference matlab-code provided by Martin Weigt. As input to the program and to Env-wide search for signature patterns we retrieved 1956 Env sequences of the Los Alamos HIV data base with a maximum of one sequence per patient and exclusion of problematic sequences, especially sequences with non-canonical amino acid symbols.

For the phylogenetic analysis and the web-logo (Figure 8) we used as the Web alignment provided at Los Alamos National Lab (http://www.hiv.lanl.gov/content/sequence/NEWALIGN/align.html. Accessed 2012 Oct 1) of HIV-1 Env, excluding problematic sequences. The alignment was then restricted to subtype B (954 sequences) and, as an outgroup, the 10 most informative sequences of subtype D. The phylogenetic tree was constructed with PhyML [47] using the HIVb model of amino acid substitution with a proportion of invariable sites and rate heterogeneity of substitution. Nearest-neighbor interchange was used for heuristic tree searches and branch support was estimated with approximate likelihood ratios. Trees were plotted with R-package ape [48].

The web-logos of non-East Asian (B) and East Asian (B') (Figure 8) V2 sequences were generated by splitting the subtype B alignment used for the phylogenetic analysis into a B and a B' part that were submitted separately to http://weblogo.threeplusone.com/create.cgi [49], [50] (Accessed 2012 Oct 3).

For comparison with rule inference we trained a random forest [51] for classifying V3 sequences into Chinese or Non-Chinese using the above V3 data set and R-package randomForest version 4.6-6 retrieved from CRAN.

## Supporting Information

Figure S1 **Phylogenetic clustering of Env533.**
(PDF)

Figure S2 **Phylogenetic clustering of Env167 and Env792.**
(PDF)

Figure S3 **Phylogenetic clustering of Env170 and Env275.**
(PDF)

Figure S4 **Phylogenetic clustering of Env275 and Env317.**
(PDF)

Figure S5 **Cladogram of Env subtype B/B' sequences including branch support p-values.**
(PDF)

Text S1 **Alignment of V3 sequences used for the derviation of signature patterns.**
(TXT)

Text S2 **R-script for the derivation of signature patterns.**
(TXT)

Text S3 **Analysis of various sources of errors.**
(PDF)
