# Correction: A Small Set of Succinct Signature Patterns Distinguishes Chinese and Non-Chinese HIV-1 Genomes

**DOI:** 10.1371/annotation/bbb4d24d-2854-4be3-ab6b-5a2ac4c5ca8b

**Published:** 2013-12-13

**Authors:** Yan Wang, Reda Rawi, Christoph Wilms, Dominik Heider, Rongge Yang, Daniel Hoffmann

The π is missing from the 'Trp in V3 signature pattern 1 could form stabilizing cation- interaction with V2' paragraph of the Results and Discussion. The symbol should appear as follows in the first portion of the paragraph below:

**Trp in V3 signature pattern 1 could form stabilizing cation-π interaction with V2.** We had marked earlier that position 317 (V3 position 22) is usually occupied by an aromatic residue, mostly F and in B’ W. Why should an aromatic amino acid interact with the positively charged region in V2 as suggested by DI (Figure 7)? This interaction could be explained by cation-π interactions between the cationic V2 region and the π-electron system of the aromatic F or W [38,39].

