# Supplementary material for: A Small Set of Succinct Signature Patterns Distinguishes Chinese and Non-Chinese HIV-1 Genomes
Source: PLoS One. 2013 Mar 19;8(3):e58804. doi: 10.1371/journal.pone.0058804 (PMC3602349; doi:10.1371/journal.pone.0058804)
Supplement: Figure S1 — Phylogenetic clustering of Env533. (PDF) [file pone.0058804.s001.pdf]

Phylogenetic clustering of Env553

–

Supplementary information for:

A small set of succinct signature patterns distinguishes Chinese and non-Chinese HIV-1 genomes

Yan Wang[1,2,†], Reda Rawi[1,†], Christoph Wilms[1], Dominik Heider[1], Rongge Yang[2,*], Daniel Hoffmann[1,*]

1 Research Group Bioinformatics, Center for Medical Biology, University of Duisburg-Essen, Essen, Germany; 2 AIDS and HIV Research Group, State key Laboratory of Virology, Wuhan Institute of Virology, Chinese Academy of Sciences, Wuhan, P.R.China;* E-mail: ryang@wh.iov.cn, daniel.hoffmann@uni-due.de

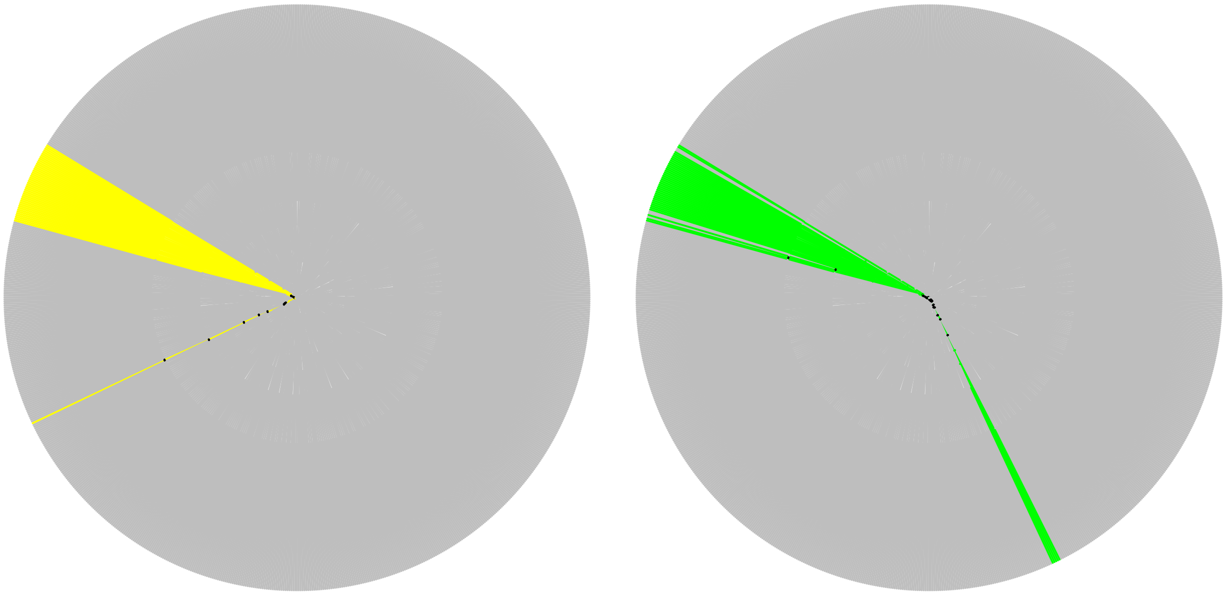

Figure 1: Phylogenetic clustering of the Env position corresponding to HXB2 Env553. The underlying fan tree is the same described in the main manuscript. Left side: branches in agreement with pattern Env553 = R colored yellow. Right side: branches of subtype B samples from China colored green. The comparison shows that Env553 = R is a good marker for Chinese sequences.
