# Supplementary material for: A Small Set of Succinct Signature Patterns Distinguishes Chinese and Non-Chinese HIV-1 Genomes
Source: PLoS One. 2013 Mar 19;8(3):e58804. doi: 10.1371/journal.pone.0058804 (PMC3602349; doi:10.1371/journal.pone.0058804)
Supplement: Figure S4 — Phylogenetic clustering of Env275 and Env317. (PDF) [file pone.0058804.s004.pdf]

Phylogenetic clustering of Env275 and Env317

–

Supplementary information for:

A small set of succinct signature patterns distinguishes Chinese and non-Chinese HIV-1 genomes

Yan Wang[1,2,†], Reda Rawi[1,†], Christoph Wilms[1], Dominik Heider[1], Rongge Yang[2,*], Daniel Hoffmann[1,*]

1 Research Group Bioinformatics, Center for Medical Biology, University of Duisburg-Essen, Essen, Germany; 2 AIDS and HIV Research Group, State key Laboratory of Virology, Wuhan Institute of Virology, Chinese Academy of Sciences, Wuhan, P.R.China;* E-mail: ryang@wh.iov.cn, daniel.hoffmann@uni-due.de

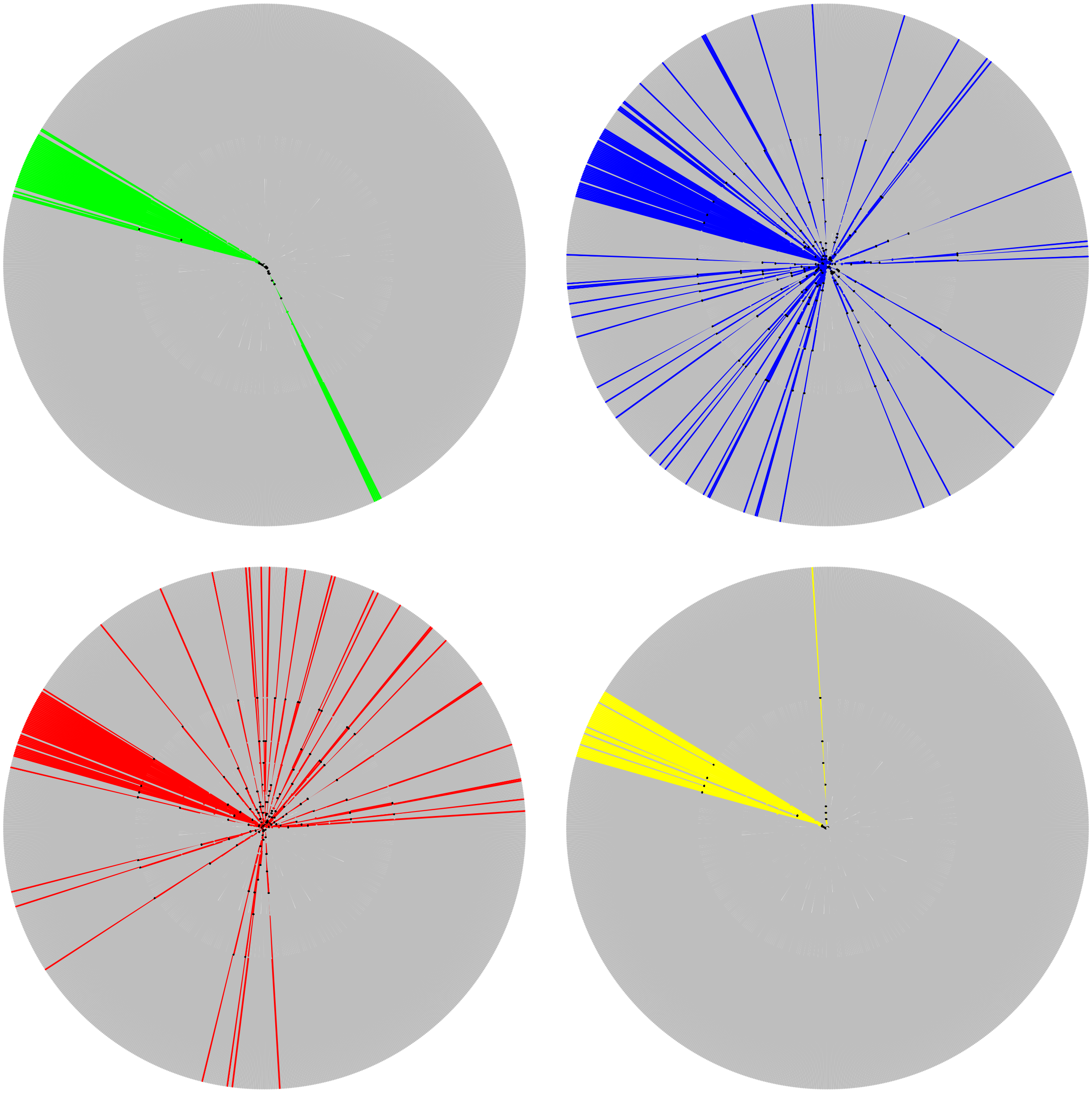

Figure 1: Phylogenetic clustering of Env positions corresponding to HXB2 Env positions 275 and 317. The underlying fan tree is the same described in the main manuscript. Top left (green): branches of subtype B samples from China. Top right (blue): branches matching pattern Env317 = W. Bottom left (red): branches matching Env275 = S. Bottom right (yellow): branches matching Env275 = S AND Env317 = W. The comparison between top left and bottom right shows that pattern Env275 = S AND Env317 = W is a good marker for Chinese sequences.
