# Supplementary figures and images for: A Small Set of Succinct Signature Patterns Distinguishes Chinese and Non-Chinese HIV-1 Genomes
Source: PLoS One. 2013 Mar 19;8(3):e58804. doi: 10.1371/journal.pone.0058804 (PMC3602349; doi:10.1371/journal.pone.0058804)

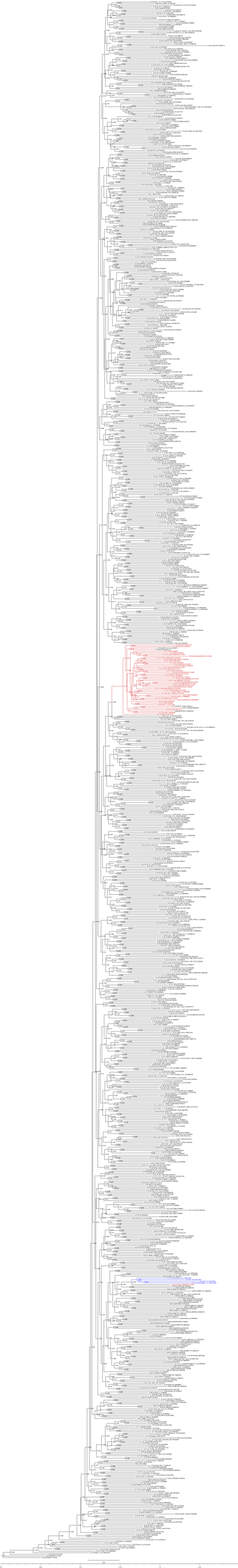

Supplement: Figure S5 — Cladogram of Env subtype B/B' sequences including branch support p-values. (PDF) [file pone.0058804.s005.pdf]
