# Supplementary material for: A Small Set of Succinct Signature Patterns Distinguishes Chinese and Non-Chinese HIV-1 Genomes
Source: PLoS One. 2013 Mar 19;8(3):e58804. doi: 10.1371/journal.pone.0058804 (PMC3602349; doi:10.1371/journal.pone.0058804)
Supplement: Text S3 — Analysis of various sources of errors. (PDF) [file pone.0058804.s008.pdf]

Error Assessment for Signature Patterns

Supplementary information for:

A small set of succinct signature patterns distinguishes Chinese and non-Chinese HIV-1 genomes

Yan Wang[1,2,†], Reda Rawi[1,†], Christoph Wilms[1],
Dominik Heider[1], Rongge Yang[2,*], Daniel Hoffmann[1,*]

1 Research Group Bioinformatics, Center for Medical Biology,
University of Duisburg-Essen, Essen, Germany;
2 AIDS and HIV Research Group, State Key Laboratory of Virology,
Wuhan Institute of Virology, Chinese Academy of Sciences, Wuhan, P.R.China;
*E-mail: ryang@wh.iov.cn, daniel.hoffmann@uni-due.de

Contents

1 Statistical errors of signature patterns 1
- 1.1 Classification performance 2
- 1.2 Robustness of signature pattern generation 3

2 Reliability of phylogeny 3

3 Structure of Chinese V3 sequence data and possible confounding factors 5
- 3.1 Co-receptor tropism as potential bias 5
- 3.2 Regional distribution as potential bias 6
- 3.3 Risk factors 6

1 Statistical errors of signature patterns

This section addresses two questions related to statistical errors of the classification with signature patterns: (1) What is the classification performance of the set of signature patterns? (2) How robustly does the rule inference algorithm generate individual signature patterns? In the following two subsections we address both questions.

1.1 Classification performance

First we assessed the performance of signature patterns in the classification of V3 sequences into classes Chinese or non-Chinese. To this end, we performed a full leave-one-out classification run with the same set of 2335 V3 sequences used in the main manuscript (1047 Chinese, 1288 non-Chinese): each of the sequences was left-out once from the training data, and a set of signature patterns was learned with RIPPER [1] from the remaining 2334 sequences and their class labels as described in the main text; this was followed by the classification of the left out sequence into Chinese or non-Chinese with this set of signature patterns. Comparison of the 2335 predicted and true class labels allowed for an assessment of the prediction performance. Figure 1 shows the Receiver Operating Characteristic (ROC) curve [2], with an area under the curve (AUC) of 0.89. This good performance is in line with the low errors reported for the set of 14 signature patterns in the main text.

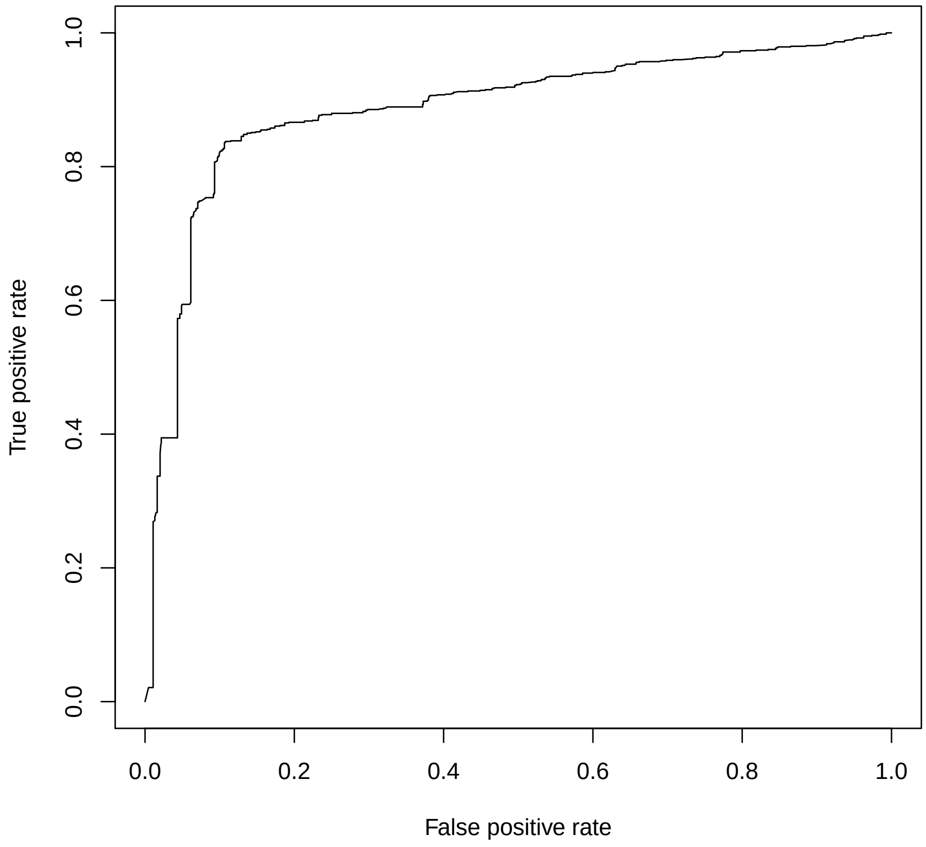

Figure 1: ROC curve from leave-one-out classification run with all V3 sequences.

1.2 Robustness of signature pattern generation

A sequence classifier trained with RIPPER [1] comprises a full *set* of signature patterns, e.g. the 14 signature patterns for V3 sequences analyzed in the main manuscript. There, we have seen that the elementary signature patterns in the set are associated with the phylogenetic structure of the data, namely the HIV subtypes. Since we do not fundamentally change the phylogenetic structure of the data in the leave-one-out experiment in subsection 1.1, we might expect that not only the sets of signature patterns performs consistently well in the classification task, but that also the same elementary signature patterns in the sets are recovered repeatedly. To test this hypothesis, we have collected all elementary signature patterns that were generated in the leave-one-out experiment in subsection 1.1, and we have counted the frequency with which each of these elementary patterns was generated. These frequencies must not be interpreted as confidence in the respective pattern (as for instance in bootstrapping): the RIPPER heuristic had been designed to yield concise *sets* of short patterns, so that a whole set of patterns forms a classifier with good performance. Thus, the algorithm systematically suppresses redundant and long patterns, even if they are statistically highly significant. Figure 2 summarizes the result. All in all, 590 different elementary patterns were generated in the 2335 iterations of the leave-one-out run. A few of the elementary patterns turned up very often, while most of the elementary patterns were obtained only rarely. Table 1.2 shows the elementary patterns that top the frequency chart. The signature pattern generated most frequently is the LW-pattern 1 analyzed in detail in the main manuscript. It occurs in 2216 of the 2335 runs, i.e. in 95% of the runs. The next pattern (single R at position 24) in the list corresponds to pattern 2 of the main manuscript, occurring in 70% of the runs, etc. Patterns 3 and 4 of the main manuscript take ranks 5 and 6, respectively, in Table 1.2, each occurring in 38% of the runs. In summary, the most powerful patterns identified by the single application of RIPPER to the V3 sequence data in the main manuscript also belong to the 1% most frequently generated patterns in the leave-one-out experiment. We also see that the patterns taking the third and fourth rank in Table 1.2 do not occur in our set described in the main manuscript, presumably because they are redundant with other patterns that are already in this set.

2 Reliability of phylogeny

In order to test the V3 signature pattern 1 (LW pattern), we have in the main manuscript applied a maximum likelihood phylogeny method to an independent set of 964 Env sequences. This phylogenetic analysis supported the strong association of the LW pattern with subtype B'. Since the phylogeny is on one hand not central to the manuscript, and on the other hand, bootstrapping of a tree of this size is computationally expensive (a single tree computation took about 200 h) and difficult to present, we have resorted to the fast approximate likelihood ratio test (aLRT) offered by PhyML [3]. Specifically, we computed the branch support from aLRT statistics using a Shimodaira-Hasegawa (SH)-like procedure [4]. The p-values obtained from the SH-test allow at a significance level of 0.05 rejection

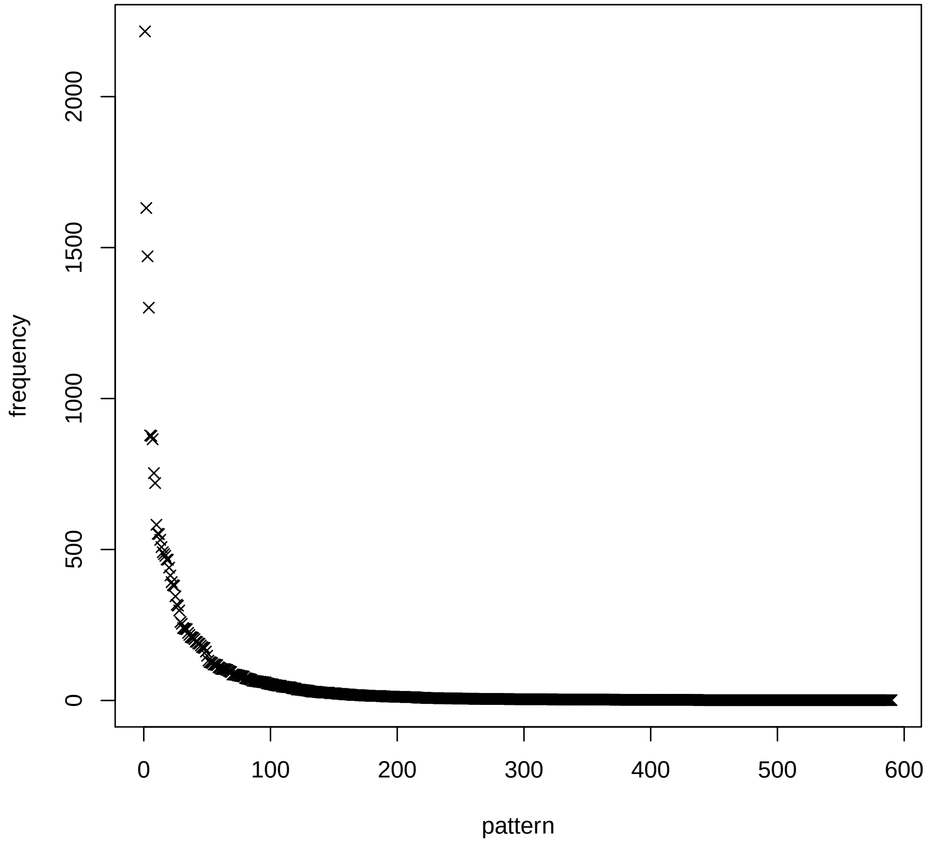

Figure 2: Frequency of generated signature patterns.

of the null hypothesis that the tested branch has length 0. The cladogram in Figure S8 (p-values as annotations) shows clearly that Chinese sequences carrying signature pattern 1 form two narrow clusters, colored red and blue, including a few other sequences from Thailand, Myanmar, and Japan.

3 Structure of Chinese V3 sequence data and possible confounding factors

The hypothesis put forward in the manuscript is essentially that the signature patterns for Chinese sequences are due to specific founder sequences, and that the patterns are possibly stabilized by interactions that maintain biomolecular function. This hypothesis is parsimonious, plausible, and in agreement with the data presented in the main manuscript. However, it is imaginable that the sequence data is strongly biased so that the patterns are a result of this bias. In the following, we present analyses that exclude several factors that could determine the patterns. As in the main manuscript, we focus on signature pattern 1. In anticipation of the results presented in the following, we can state that we could not find a bias that would lead to the rejection of the above hypothesis.

3.1 Co-receptor tropism as potential bias

It could be that V3 sequences conforming to signature pattern 1 are taken from CCR5-tropic virus, while sequences not conforming to the pattern are from CXCR4-tropic virus (or vice versa). The main problem here is that we have experimental tropism information for only 219 (158 R5-tropic, 61 X4-tropic) of the 1047 Chinese V3 sequences used for rule inference. We have therefore resorted to using tropism computationally predicted by TCUP2 (Two-Level Co-receptor Usage prediction 2), the successor of TCUP published in Ref. [5]. We have first tested the prediction performance of TCUP2 on the 219 Chinese sequences with given tropism information. In a patient-wise leave-one-out on the Chinese sequences, the AUC was 0.96. Using a cutoff probability of 0.48, we achieved a sensitivity of 0.72 and a specificity of 0.97. Given these results, we were confident that TCUP2 will predict tropism of the Chinese V3 sequences with good accuracy. We submitted to TCUP2 all 1047 Chinese sequences for prediction of co-receptor tropism to with TCUP2. (For three of the 249 sequences that show signature pattern 1, TCUP2 did not yield a prediction because of missing Cys at a V3 terminus, i.e. the violation of one of the filter criteria of TCUP2). The predicted tropisms were then used to test the hypothesis that tropism can explain signature pattern 1 (see Table 2 on page 8). When applying Fisher's exact test to Table 2, we find a p-value of 0.11. Assuming a significance level of 0.05, we have no significant association of signature pattern 1 and tropism. Tropism cannot explain signature pattern 1.

3.2 Regional distribution as potential bias

Another theoretical explanation of the signature patterns is that sequences found to fulfill a certain pattern could have been collected from a local outbreak in China. If that would be the case, we should expect a geographical distribution with a strong center and much smaller contributions from other parts. As a simple check, we have therefore collected information on the provinces of origin of the V3 sequences that carry signature pattern 1. Table 3 on page 8 shows the result. According to the table, this pattern is found in samples from many provinces, and thus there is no evidence for the origin of signature pattern 1 in a recent local outbreak.

It is important to remember that while we have no evidence for an origin of the pattern in a recent outbreak, there is evidence for the local origin of subtype B' many years ago (see Ref. [6]), and thus also for signature pattern 1, which has been found to be highly significantly associated with subtype B' as described in the main manuscript.

3.3 Risk factors

In the main manuscript, we have shortly recapitulated what has been found out about the genesis and spread of subtype B'. Table 3.3 seems to support this history. It is important to note that for most of the sequences we could not extract information on the risk factors (see column "NA"). However, this does not affect our conclusions reported in the main manuscript.

References

[1] William W. Cohen. Fast effective rule induction. In *In Proceedings of the Twelfth International Conference on Machine Learning*, pages 115–123. Morgan Kaufmann, 1995.

[2] T Sing, O Sander, N Beerenwinkel, and T Lengauer. Rocr: visualizing classifier performance in r. *Bioinformatics*, 21(20):3940–1, Oct 2005.

[3] S Guindon, F Delsuc, J F Dufayard, and O Gascuel. Estimating maximum likelihood phylogenies with phyml. *Methods Mol Biol*, 537:113–37, 2009.

[4] H Shimodaira and M Hasegawa. Multiple comparisons of log-likelihoods with applications to phylogenetic inference. *Molecular Biology and Evolution*, 16(8):1114, 1999.

[5] J N Dybowski, D Heider, and D Hoffmann. Prediction of co-receptor usage of hiv-1 from genotype. *PLoS Comput Biol*, 6(4):e1000743, Apr 2010.

[6] X Deng, H Liu, Y Shao, S Rayner, and R Yang. The epidemic origin and molecular properties of b': a founder strain of the hiv-1 transmission in asia. *AIDS*, 22(14):1851–8, Sep 2008.

Table 1: The 30 signature patterns that were generated most frequently in the leave-one-out run. Positions in patterns correspond to columns in alignment of V3 sequences described in main manuscript; the first row shows the aligned HXB2 reference sequence for orientation. Dots stand for unspecified positions in the respective patterns. Letters and hyphen have their usual meaning in sequence alignments, i.e. amino acids and gaps, respectively. Frequencies are given in the right column.

no.	pattern	frequency
HXB2	CTRPNN-NT-R-KRIR---IQRG-PGR--A-FVTIG-----KIG---NMRQAHC	
1	...................L...........W......................	2216
2	.................................R....................	1631
3	...............Y........................R.............	1471
4	..........................R..S........................	1301
5	..............I...........Q..T.....G..................	878
6	............K.I...........Q..T.....G..................	876
7	..............I...........Q.............Q.............	865
8	.............................S.L......................	753
9	............T.I...........Q.......................K...	720
10	...............................W..................Q...	582
11	............K.............Q......A......-.............	552
12	....S........................V........................	551
13	............K......I...........W..................Q...	532
14	............K................T...A..................Y.	508
15	............K.............Q..T......................Y.	491
16	....S.......T...	485
17	............K.IR.............T.....G..................	479
18	..............I...........Q........E..................	467
19	.T..........K.IR..........Q.............D.............	466
20	.............................V....................K...	440
21	....G.........I...........Q..T........................	414
22	............K..................L........Q.............	392
23	.............................V.................D..K...	382
24	...............................F.R....................	380
25	..........................Q........E..................	345
26	....S........................V.F......................	317
27	.......N.........................R....................	314
28	...............................W........Q.............	298
29	...............................L........Q.............	260
30	............K.V..............T...A..................Y.	254

	pattern 1	not pattern 1
X4-tropic	14	72
R5-tropic	232	725

Table 2: Contingency table for fulfilment of signature pattern 1 by 1047 Chinese V3 sequences vs. tropism.

Province	-	BJ	CNE	GX	HA	HB	HLJ	HN	HW	JS	LN	SHX	YN
Frequency	14	14	14	4	37	11	9	41	8	1	11	33	52

Table 3: Chinese provinces of origin of V3 sequences conforming to signature pattern 1. "-" means that no province could be assigned.

Risk factor	MB	NO	NR	PB	PI	SH	SM	SU	SW	NA
Count (all)	1	21	17	19	56	55	36	5	1	836
Count (pat. 1)	-	17	-	13	5	3	1	-	-	201

Table 4: Risk factors associated with all Chinese V3 sequences analyzed (first row), and risk factors reported for sequences carrying signature pattern 1 (second row). Risk factors are: SG - homosexual; SB - bisexual; SM - male sex with male; SH - heterosexual; SW - sex worker; SU - sexual transmission, unspecified type; PH - hemophiliac; PB - Blood transfusion; PI - IV drug use; MB - Mother-baby; NO - Nosocomial; EX - Experimental; NA - not recorded (or unknown); OT - other.
